# Multi-scale modelling of the dynamics of cell colonies: insights into cell-adhesion forces and cancer invasion from *in silico* simulations

**DOI:** 10.1098/rsif.2014.1080

**Published:** 2015-02-06

**Authors:** Daniela K. Schlüter, Ignacio Ramis-Conde, Mark A. J. Chaplain

**Affiliations:** 1 Division of Mathematics, The University of Dundee, Dundee, UK; 2 Department of Mathematics, Faculty of Education, Universidad de Castilla la Mancha, Cuenca, Spain

**Keywords:** individual-based modelling, cell population modelling, cell-adhesion forces, E-cadherin

## Abstract

Studying the biophysical interactions between cells is crucial to understanding how normal tissue develops, how it is structured and also when malfunctions occur. Traditional experiments try to infer events at the tissue level after observing the behaviour of and interactions between individual cells. This approach assumes that cells behave in the same biophysical manner in isolated experiments as they do within colonies and tissues. In this paper, we develop a multi-scale multi-compartment mathematical model that accounts for the principal biophysical interactions and adhesion pathways not only at a cell–cell level but also at the level of cell colonies (in contrast to the traditional approach). Our results suggest that adhesion/separation forces between cells may be lower in cell colonies than traditional isolated single-cell experiments infer. As a consequence, isolated single-cell experiments may be insufficient to deduce important biological processes such as single-cell invasion after detachment from a solid tumour. The simulations further show that kinetic rates and cell biophysical characteristics such as pressure-related cell-cycle arrest have a major influence on cell colony patterns and can allow for the development of protrusive cellular structures as seen in invasive cancer cell lines independent of expression levels of pro-invasion molecules.

## 1. Introduction

Traditional experiments for understanding the influence of cell adhesion on tissue structure may be classified into two principal groups. Firstly, experiments that study the adhesive behaviour of cell colonies *in vitro* or *in vivo* [1–3], and secondly, isolated cell experiments where the inter-cellular forces are measured via micro-pipette assays [4]. While the colony behaviour as a whole can be observed in cell colony experiments, micro-pipette assays obtain information concerning the behaviour of one or two isolated cells. The question then arises whether conclusions can be drawn and extrapolated to cellular behaviour at the colony level from the forces measured in isolated cell assays.

To approach this question, we propose a multi-scale—multi-compartment model that captures the biophysical essentials of the cell-adhesion system and relates intracellular and intercellular phenotypic characteristics to cell culture systems. The cell–cell interactions are modelled using a potential function which leads to a force-based model. The repulsive forces between two cells are governed by their bio-mechanical properties, and the strength of adhesion between two cells is determined by the concentration of E-cadherin–*β*-catenin bonds they form. The model is developed by testing simulation results of various hypotheses for the explicit structure of the adhesion pathway and cell–cell interactions against given cell colony data. This allows us to find the basic interactions necessary for realistic cell colony development. The intra- and intercellular adhesion pathway is modelled using ordinary differential equations and a dynamic compartmentalization of the cell's intracellular domain in terms of cell–cell contact areas. This new approach allows us to understand the adhesive behaviour of each set of neighbouring cells concomitantly. In contrast to the earlier model developed by Ramis-Conde *et al.* [5], including compartmentalization provides for spatial heterogeneity of the adhesion proteins. This new feature is essential to understand whether it is plausible to extrapolate conclusions from isolated cell experiments to the cell colony level as it allows us to compare cell–cell adhesion forces between individual cells in a colony with forces measured between pairs of isolated cells in micro-pipette assays.

### 1.1. The E-cadherin–β-catenin adhesion pathway

E-cadherins are calcium-dependent proteins of the cell–cell adhesion system. They play a principal role in the formation of junctional contacts between cells and are essential for the proper functioning of many biological processes. Under-expression of adhesion molecules or malfunctioning of the cadherin adhesion system has been linked directly to many diseases including metastatic cancer [6].

Following adhesion pathway activation, E-cadherin and *β*-catenin bind at the endoplasmatic reticulum immediately after production [7]. The complex is then trafficked to the cell membrane [7,8]. This transport takes place either in a directed manner with the purpose of the complex being exocytosed at a specific cell–cell contact site, or undirected leading to E-cadherin–*β*-catenin complexes distributed across the entire cell surface, also on non-adherent cells [9]. Once positioned at the membrane, the complex can thus either remain isolated and form part of a pool of molecules, which are dynamically endocytosed and either recycled and transported back to the cell surface or degraded, or it can undergo a series of transformations that ultimately will create an effective bond [9]. At the intracellular level, these transformations include the binding of molecules of the catenin family, which help to form a scaffolding machinery that connects the extracellular domain of the adhesive complex with the cells cytoskeleton [10]. The extracellular E-cadherin tails form dimers that bind to their homologues on neighbouring cells.

Despite the fact that E-cadherins form stable bonds between cells, the adhesion pathway is not a stationary system [9]. The E-cadherin–*β*-catenin complex can be constantly endocytosed, recycled to create bonds at different positions or degraded into smaller molecules [11]. The active dynamics determine the cell–cell adhesive forces and tissue architecture.

## 2. The model

We develop a multi-scale model of cell–cell interactions. This model encompasses E-cadherin–*β*-catenin dynamics which are governed by kinetic rates as well as changes in cell–cell contact areas. The bond concentration between two cells at a cell–cell contact site is then used to calculate the adhesion/separation force. In addition, we calculate cell–cell repulsive forces using the Hertz model [12,13].

### 2.1. Adhesion pathway equations

In our model, a cell consists of a set of compartments between which the molecules are trafficked depending on the adhesion dynamics. Each cell–cell contact site, as well as the cell's cytoplasm, is considered as a dynamic separate compartment. Free E-cadherin and free *β*-catenin only exist in the cytoplasmic compartment. The cytoplasmic compartment can also hold E-cadherin–*β*-catenin complexes which includes those complexes that are at the cell membrane but not at a site of cell–cell contact. E-cadherin–*β*-catenin complexes at a specific contact site are in the corresponding cell–cell contact compartment.

We model the dynamics described above by taking into account E-cadherin–*β*-catenin complex formation, directed and undirected transport and exocytosis at the cell membrane, endocytosis and complex dissociation. We assume that only those complexes that are not involved in adherens junctions are endocytosed [9] or endocytosed junctional complexes are replaced almost immediately by exocytosis of a newly formed complex. Endocytosis also occurs after adherens junction disassembly.

**Figure 1.**
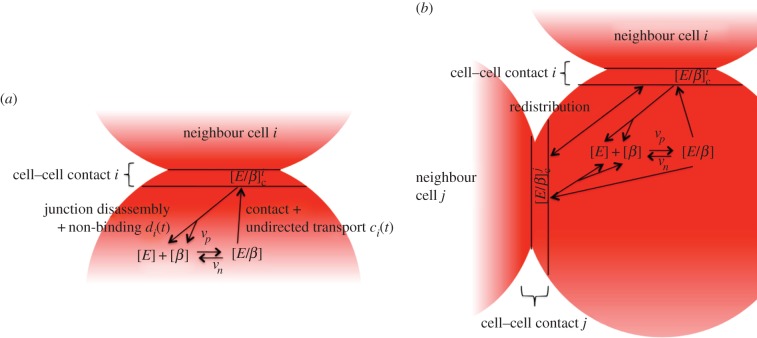
Schematic diagram showing the E-cadherin–*β*-catenin dynamics as considered in the Model 1 [(*a*)] and Model 2 [(*a*) + (*b*)]. Free E-cadherin (*E*) and *β*-catenin (*β*) in the cytoplasm bind to form a complex (*E*/*β*). In adherent cells, in addition to the general transport to the cell surface, E-cadherin–*β*-catenin complexes are trafficked to the contact area. If the complex is transported to a site of cell–cell contact (denoted 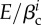 at cell contact site *i*), it can bind complexes on the neighbouring cell's surface. If there is no binding partner, the complex can be internalized again and recycled. The same process takes place when bonds are broken due to junction disassembly. Whereas in Model 1, the amount of E-cadherin–*β*-catenin complexes that can be trafficked to one cell–cell contact site is limited, Model 2 comprises of additional dynamics such that the E-cadherin–*β*-catenin complexes are redistributed between contact sites when a cell has got more than one neighbouring cell as is shown in (*b*). (Online version in colour.)

Endocytosis is followed by the disruption of the E-cadherin–*β*-catenin complex and the components can either be degraded, recycled for cell–cell adhesion or re-used in different signalling contexts. As production of neither E-cadherin nor *β*-catenin is explicitly taken into account, the possibility of degradation is also not considered in the model but it is assumed that the overall number of molecules is at a steady state. The model dynamics are shown in figure 1.

The equations governing these E-cadherin–*β*-catenin dynamics are given as follows:2.1
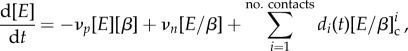
2.2
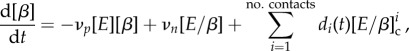
2.3
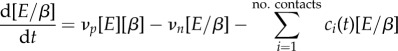
2.4
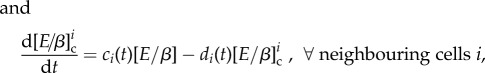
where [*E*] is free E-cadherin, [*β*] is free *β*-catenin, [*E*/*β*] are non-adhesion effective E-cadherin–*β*-catenin complexes in the cytosol or at the cell membrane at non-contact sites and [*E*/*β*]*^i^*_c_ are E-cadherin–*β*-catenin complexes at the contact site with cell *i*. *ν_p_* is the E-cadherin–*β*-catenin binding rate and *ν_n_* the complex dissociation rate. *d_i_*(*t*) describes the endocytosis of E-cadherin–*β*-catenin complexes due to junctional disassembly at contact site *i*. Thus2.5
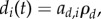
where *ρ*_*d*_ is a complex translocation rate and *a*_*d,i*_ gives the loss of contact area with cell *i* at time *t*:
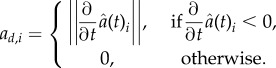
Here, 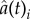 is the contact area between the two cells at time *t*. This term for the internalization due to junctional disassembly is taken from [5].

Similarly, *c_i_*(*t*) describes the exocytosis of E-cadherin–*β*-catenin complexes at the site of contact with cell *i*.

#### 2.1.1. Two hypotheses for adhesion complex exocytosis

We consider two biological hypotheses involved in the exocytosis of adhesion complexes. In both hypothesis, we assume that two principal processes are involved: (i) directed exocytosis: after cell membrane stimulation, the E-cadherin–*β*-catenin complexes are transported from the cytosol to the specific area of cell–cell contact to create an effective bond; and (ii) undirected transport: complexes are transported and exocytosed at arbitrary places on the cell surface.

##### 2.1.1.1. Model 1: static adhesion hypothesis

The first hypothesis assumes that the adhesion between two cells is static, while the size of their contact area remains unchanged. This implies that after an effective bond has been created, it will not be broken unless the complexes involved are immediately replaced and the bond is reformed such that the adhesion structure remains unchanged. In order for multiple cells to be able to adhere to a cell, the number of complexes per contact area therefore has to be limited. The schematic diagram in figure 1*a* shows the principal interactions considered by this hypothesis. We model this by including the following exocytosis term:2.6
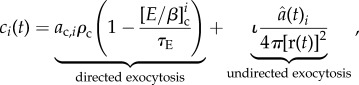
where *ρ*_c_ is the E-cadherin–*β*-catenin complex translocation rate from the cytosol to the cell–cell contact location, *τ*_E_ = *E*_t_/6 is the maximum concentration of adhesion complexes per effective bond and *E*_t_ is the total E-cadherin concentration within the cell. The directed exocytosis term is dependent on the increase in contact area given by
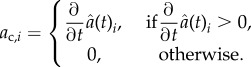
The contribution from undirected transport is proportional to the ratio of the contact area to the cell surface area with factor 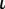.

##### 2.1.1.2. Model 2: dynamic adhesion hypothesis

In the second hypothesis, we assume that effective adhesion complexes at the cell membrane can be directly re-organized to form bonds at different locations by a transport process within the cell membrane [9]. This implies that almost the total number of possible E-cadherin–*β*-catenin complexes can be employed at a single cell–cell contact site, but as soon as contact is made with another cell, the complexes are redistributed. Figure 1*b* shows a schematic diagram of the adhesion dynamics using this hypothesis. In this case, the exocytosis term is given by2.7
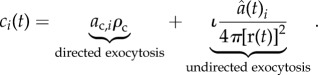
The redistribution of E-cadherin–*β*-catenin complexes between different cell–cell contact sites is modelled by the following equations:2.8
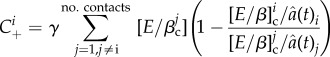
and2.9
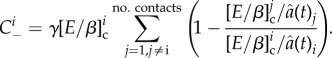


Here, 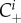 is the number of E-cadherin–*β*-catenin complexes that are translocated from other cell–cell contact sites to the site of contact with cell *i*, and 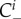 is the number of complexes that is moved from cell–cell contact site *i* to other contact sites. In both equations, the sum is only taken over those terms for which the term in the bracket is greater than zero. 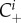 is added and 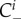 is subtracted from the right-hand side of equation (2.4) in Model 2 giving the equation2.10
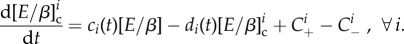


### 2.2. Cell–cell interactions

We approximate cells as visco-elastic spheres [5,13–15], and model the repulsive–adhesive interactions by the extended Hertz model used by Ramis-Conde *et al.* [5,14], Hertz [12] and Landau & Lifschitz [16]. The potential *V_ij_* that arises from these interactions is calculated as follows:2.11
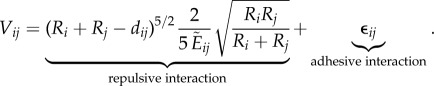


The first term on the right-hand side is the repulsive interaction given by the Hertz model with
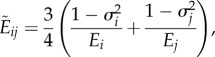
where *d*_*ij*_ is the distance between the centres of the two cells, *σ*_*i*_ and *σ*_*j*_ are the Poisson ratios of the spheres and *E*_*i*_ and *E*_*j*_ are the elastic moduli. *R*_*i*_ and *R*_*j*_ are the radii of cell *i* and *j*, respectively. The force **F**_*ij*_ acting on the cell due to interactions with its neighbours is given by the negative derivative of the potential2.12
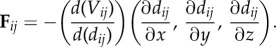
As we consider the E-cadherin pathway explicitly and therefore know the number of E-cadherin bonds at the individual cell–cell contact sites and can couple them with experimental force measurements, we derive the adhesion term for the force equation (2.12) rather than for the equation of the potential (2.11). The cell–cell adhesive forces are governed by the E-cadherin–*β*-catenin complexes on the surface of two neighbouring cells at the contact site. The smaller number of complexes on either cell's surface at the contact site determines how strong the adhesion is, as it limits the number of bonds that can be formed. This number of E-cadherin–*β*-catenin complexes involved in cell–cell adhesion bonds is given as a percentage of the maximum number of complexes that can theoretically be formed (=100%). The number of bonds between two cells is translated into a cell–cell adhesion force by using the experimental data by Chu *et al.* [4]. Assuming that each cell in a monolayer should be able to have six neighbours and some free E-cadherin and *β*-catenin in the cytosol, in Model 1 only about 15% of the total possible number of complexes can be found at a cell–cell contact site. Thus for this model, we assume that in the given data from [4] only 15% of all possible E-cadherin–*β*-catenin complexes are at the contact site and generate the measured separation force of 210 nN. For Model 2, we assume that 80% of the possible E-cadherin–*β*-catenin complexes in a cell produce the force of 210 nN. Thus, the adhesion force between two cells in the two models is calculated as follows:2.13
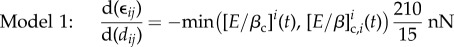
and2.14
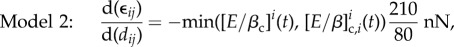
where 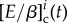 is the percentage of E-cadherin–*β*-catenin complexes at the site of contact with cell *i* expressed by the cell currently of interest and 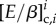 is the percentage of E-cadherin–*β*-catenin complexes at the site of contact with cell *i*, expressed by cell *i*.

#### 2.2.1. Separation force framework

We further develop a second variant of the cell–cell interaction model by including an artificial constraint to cell–cell adhesion forces. Instead of considering E-cadherin–*β*-catenin bonds as constantly active adhesive bonds, they are assumed to merely prevent separation. This is done to reflect the fact that forces are measured in experiments during separation processes rather than in a static adhesion setting. We assume that cells in monolayers have cell–cell contact areas, the diameters of which are one-sixth of the cell's circumference and we call this the cell's ‘natural state’. Cells in their natural state or closer than that are assumed to not actively adhere to one another. Thus, the adhesion force is zero and therefore the overall force equals the repulsive force alone. In the case of Model 2, the dynamic adhesion model, this leads to the following adhesion term:2.15
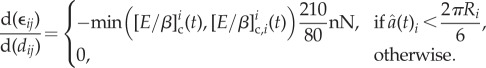
Although merely a modification, we will refer to this equation in conjunction with equations (2.7)–(2.10) as Model 3.

### 2.3. Cell division

We model the cell cycle by assuming that the G1-phase has an average length of 7 h. During the G1-phase, a cell grows uniformly up to its maximum radius *R*. The M-phase has an average length of 2 h; and G0, S and G2 together have a length uniformly distributed between 8 and 18 h. This gives an overall cell-cycle length of 17–27 h [15,17]. Cell division occurs along the axis of highest pressure resulting in two new cells of radius *R*/2^1/3^ preserving volume conservation [5]. During the M-phase, cells have a dumb-bell shape up to division. This is modelled by suppressing cell–cell interactions between the two daughter cells during this phase.

Whenever explicitly stated, we additionally take into account contact inhibition of proliferation [18,19]. In this case, we assume that if the cell is exerting a repulsive force above a certain threshold, the cell's division cycle is paused and it does not enter M-phase. The threshold force chosen is 13 000 pN which translates to a cell having six neighbours each with a distance of ≈ 8.5 µm from cell-midpoint to cell-midpoint.

### 2.4. Cell movement

The cell movement is governed by the total force acting on the individual cell [5,13,14]. As well as the cell–cell interactions, we take the drag force and the random movement into account. Thus, the equation of motion is2.16
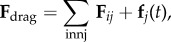
where **F***_ij_* is the force generated between two cells *i* and *j* with the sum taken over all cells that cell *j* is in contact with and **f***_j_*(*t*) is the term accounting for noise. The noise term is uncorrelated and has zero mean. The drag term is calculated using Stokes' law2.17
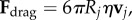
where *R_j_* is the radius of cell *j*, *η* is the viscosity of the medium and **v***_j_* is the velocity of cell *j*.

## 3. Parameter estimation

Table 1 summarizes the parameter values used in the simulations at the cell level.

**Table 1.** Table showing parameter values used to calculate the cell–cell interactions and the cell movement.

parameter		value	reference
radius of a cell	*R*	5 µm	[5,13]
Poisson ratio of cell *i*	*σ*_*i*_	1/3	[5,13]
elastic modulus of cell *i*	*E*_*i*_	1 kPa	[5,13]
suspension viscosity	*η*	10^2^ Poise	[20]

In order to parametrize the intracellular equations, the model is fitted to adhesion/separation force data taken from [4] as well as to data from growing epithelial colonies by Gibson *et al*. [21] and Sanderius *et al*. [22]. Chu *et al.* [4] have measured the separation force of two cells using a dual-pipette assay. As well as studying the influence of different E-cadherin expression levels (100, 58, 41, 38, 14 and 2%) on the force, they have also looked at the time course of the force during early cell–cell contact until its maturation after about 60 min. We use the force measurements after 5, 10, 30 and 60 min to fit the models. We further fit the models to the measurements for different E-cadherin expression levels which were taken 30 min after the initial cell–cell contact.

In the model simulations, we translate the percentage of E-cadherin–*β*-catenin bonds between two cells into an adhesion force as shown in equations (2.13) to (2.15). We then use the inbuilt MATLAB optimization routine ‘*fminsearch*’ to estimate the E-cadherin–*β*-catenin kinetic rate parameters which lead to the best fit between the experimental force measurements shown here and the forces calculated during simulations.

**Figure 2.**
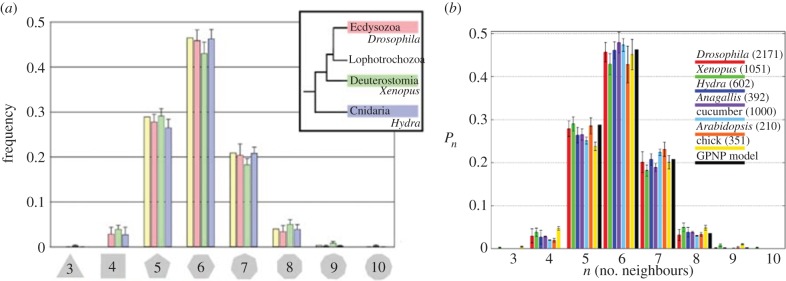
Graphs showing the distribution of the number of neighbours of cells in growing epithelial layers. (*a*) The distribution of the number of neighbours in the proliferating metazoan epithelia of *Drosophila*, *Xenopus* and *Hydra* (adapted from [21]). (*b*) The distribution of the number of neighbours of cells in a much broader range of proliferating epithelia. In addition to the epithelia of *Drosophila*, *Xenopus* and *Hydra*, it also shows the distribution for epithelia of *Anagallis*, cucumber, *Arabidopsis* and chick (also the results of a model presented in that paper are shown in black). Adapted from [22] in accordance with the Creative Commons Attribution Licence. (Online version in colour.)

In addition to the data by Chu *et al.* [4], we also use data from [21,22] (figure 2) showing the distribution of the number of neighbouring cells in a proliferating epithelial layer, to further parametrize the model. It can be seen in both figures that the distributions are very similar for this large variety of species right across the range of the Metazoa. Thus, it is a very stable pattern and we assume that it is very similar in humans as well. Rather than just the interaction of a few cells, simulations of whole cell populations are used in this case to fit the model to the data.

## 4. Results

First, we assessed whether the static or dynamic adhesion hypothesis could produce simulation results that are in agreement with the given adhesion data. Using the appropriate pathway model, we then investigated cell colony development and the effects of different cell characteristics on it using the multi-scale model. The initial conditions for the intracellular species in the simulations are as follows: [*E*](0) = % E-cadherin expression level (here generally 100), [*β*](0) = 100, [*E*/*β*](0) = 0 and 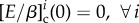.

### 4.1. Static versus dynamic adhesion

First, we tried to identify the adhesion mechanisms involved when three or more cells come into contact in a monolayer. For this, we investigated which of the two proposed models for this process, Model 1 or Model 2, could produce a better fit to the experimentally observed data [4]. To this end, we ran simulations with both model variants in which we initially placed cells next to each other and followed the intracellular dynamics for 100 min of real time. The time course of the force between two cells was noted.

**Figure 3.**
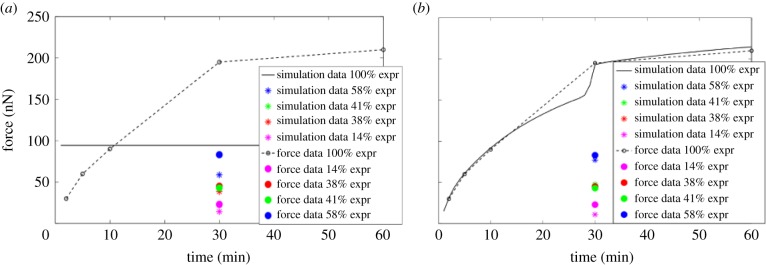
Graphs showing the results of the simulations for Model 1 and Model 2 that fit the data from [4] best. The simulation was run with both models for all the different E-cadherin expression levels considered in the experiments [4]. The whole time course is shown for simulation with 100% E-cadherin expression as multiple time points are given in the data. For lower expression levels, the measured force is only given at 30 min and thus we extract the force from the simulations at this time point only. Figure (*a*) shows the best fit for Model 1 to the data and figure (*b*) shows the best fit for Model 2 to the data. (Online version in colour.)

Initially, we assumed that only two cells come into contact. The radii of both cells were set to 5 µm and the initial total contact area between the two cells was set to 1 µm^2^. The intracellular parameters were assigned numbers randomly generated by the inbuilt MATLAB random number generator ‘*rand*’ from the log space between 10^−6^ and 10^6^. As only the attachment of two cells was considered in this round of fitting, only the parameters for the undirected complex translocation rate (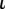), the complex dissociation rate (*ν*_*n*_), the complex formation rate (*ν*_*p*_) and the directed complex translocation rate (*ρ*_c_) could be estimated for each model. Figure 3 shows the simulation results of both models using the optimal parameter set.

Model 1 (figure 3*a*) produced a very poor fit to the force data. However, Model 2 (figure 3*b*) produced cell–cell adhesion forces that fit the data well. The parameter values found are given as the first four entries in table 2.

**Table 2.** Table showing parameter values used in the final version of the intracellular E-cadherin–*β*-catenin model. The given values were estimated to fit the model to experimentally obtained separation forces given by Chu *et al.* [4] as explained in the text.

parameter		value (min^−1^)
undirected *E*/*β* translocation rate	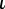	8.2
*E*/*β* dissociation rate	*ν*_*n*_	0.6
*E*/*β* binding rate	*ν*_*p*_	0.02
directed *E*/*β* translocation rate	*ρ*_c_	0.6
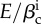 redistribution rate	*γ*	0.16

In order to find an optimal value for the redistribution rate of complexes between contact sites (*γ*), the optimization routine from above was applied to simulations of Model 2 with three cells. For the fitting, we assumed that if all three cells come into contact at the same point in time, the adhesion force should develop in the same way at both contact sites of a cell and that the adhesion force should reach half the force measured for two cells in contact by Chu *et al*. [4]. Furthermore, we assumed that if initially only two cells were in contact and then a third cell came into contact with the cell of interest, the E-cadherin–*β*-catenin complexes should be redistributed between the two contact sites, such that after ≈30 min the adhesion forces would be equal between the cell of interest and both of its neighbouring cells. The time frame was chosen in accordance with the separation force time course data by Chu *et al*. [4] which shows that the force between two cells is almost at steady state 30 min after initial contact. We assume this to be the same for the redistribution process.

**Figure 4.**
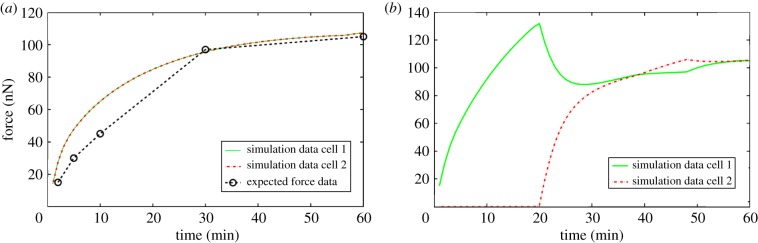
Graphs showing the time course profiles of the forces at two contact sites with two different neighbours. Figure (*a*) shows the forces over time in the case where the two cells come into contact with the cell of interest at the same time. The expected force at both contact sites is taken to be half of the force measured by Chu *et al.* [4]. Figure (*b*) shows the forces over time in the case where initially only one cell contacts the cell of interest and after 20 min a second cell comes into contact with the cell. The E-cadherin–*β*-catenin complexes are redistributed quickly so that the force at both contact sites is very similar after 10 min and completely the same after 40 min. (Online version in colour.)

Figure 4 shows the results of the simulations of Model 2 with three cells using the optimal value for the redistribution rate which is given in table 2.

Figure 4*a* shows the adhesion force between a cell and both its neighbours which came into contact with the cell at the same point in time. It can be seen that the adhesion force generated by the cell in the simulations fits the assumed actual force well. Figure 4*b* shows the development of the adhesion force between a cell and both its neighbours where initially only one of the neighbouring cells is in contact with the cell of interest, and a strong adhesion force develops between the two cells. After 20 min, the second cell comes into contact with the cell of interest and the adhesion between the initial two cells decreases, whereas the adhesion between the cell of interest and the new neighbour increases until the force at both adhesion sites is equal after about 40 min.

These investigations, which showed that results from simulations of Model 2 fit experimental data better than those of Model 1, highlight that the adhesion mechanisms in multi-cell colonies may be more complex than can be inferred by two-cell experiments. This also implies that the resulting adhesion forces in cell monolayers and tissues are below those measured through micro-pipette assays and similar techniques.

The parameter values found through the investigations are summarized in table 2. These values will be used in the following.

Further simulations showing the resulting model dynamics both, during a variety of attachment and detachment scenarios and as part of a multi-scale model, are given in the electronic supplementary material.

### 4.2. The influence of E-cadherin endocytosis on cell colony development

The only process of the intracellular model, the rate of which is still undetermined, is the E-cadherin–*β*-catenin complex endocytosis. This parameter describes the rate at which complexes are internalized after bond breakage due to the separation of two cells and might thus be important in controlling reattachment processes. Its effect can only be seen in simulations where dynamic detachment processes take place. For this reason, we ran simulations of the full multi-scale model varying this parameter by five orders of magnitude initially, before starting a more refined parameter search. We examined the simulation results after 3 days and after 7 days. To evaluate the results, we took note of the number of cells at these two time points, the number of neighbours each cell had and the average adhesion force at the cell–cell contact sites. Unfortunately, we did not have any specific cell colony data to compare the results with. However, some fundamental assumptions allowed us to compare the simulation results and decide on a reasonable parameter value.

Firstly, we assumed that the cell colony should grow considerably between day 3 and day 7. Secondly, we assumed that the cell colony should have a near-circular shape. Thirdly, we used histograms, which show the distribution of the number of neighbours of cells in a proliferating epithelial layer (figure 2), to compare the results with.

The results, first after 3 days and then after 7 days, are shown in tables 3 and 4. Rows that do not have any entries represent simulations that failed due to the cells getting so close that they could not be individually identified anymore. The colours of the cells in the plots in the second column (see online version) of the table are related to the amount of free E-cadherin as the intensity of the yellow colour in the otherwise red cells is proportional to the amount of E-cadherin in the cytosol.

**Table 3.** Table showing the results of varying the endocytosis rate *ρ*_*d*_ in terms of the cell colony development after 3 days. (Online version in colour.)

*ρ*_*d*_	image	no. cells	no. neighbours	average force (pN)
behaviour after 3 days				
500	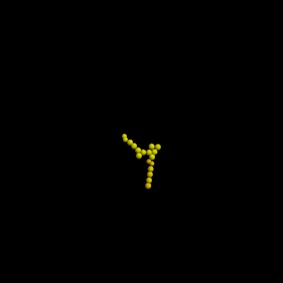	18	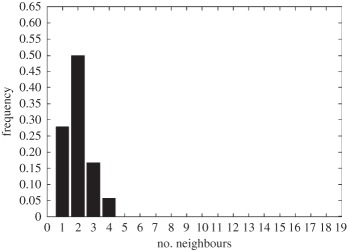	0
50	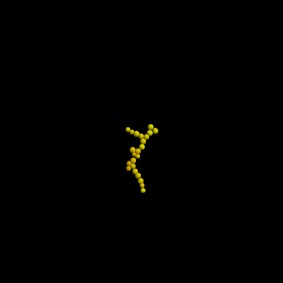	23	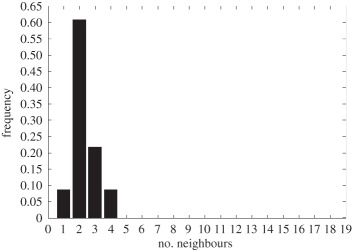	13502.69
5	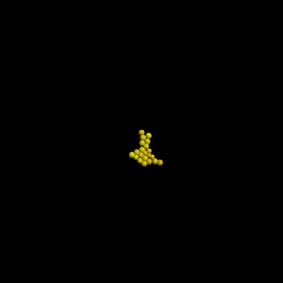	22	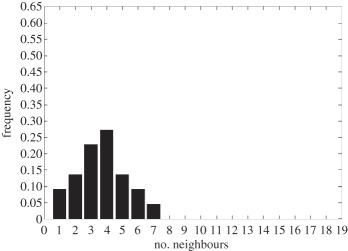	4781.9
0.5	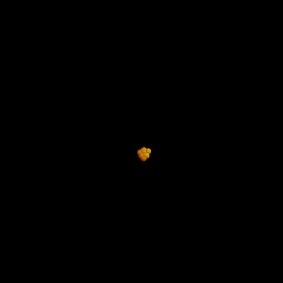	19	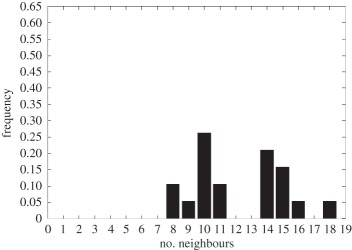	7895.95
0.05	—	—	—	—
0.005	—	—	—	—

**Table 4.** Table showing the results of varying the endocytosis rate *ρ*_*d*_ in terms of the cell colony development after 7 days. (Online version in colour.)

*ρ*_*d*_	image	no. cells	no. neighbours	average force (pN)
behaviour after 7 days				
500	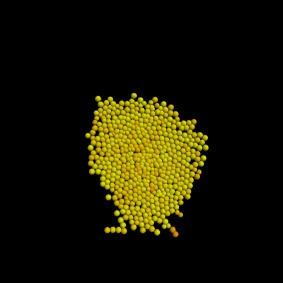	552	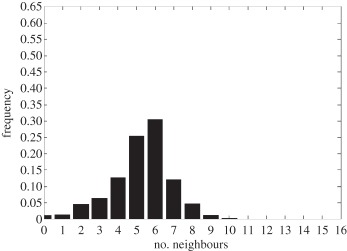	4531.67
50	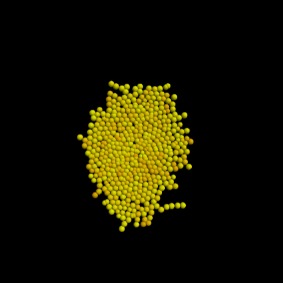	528	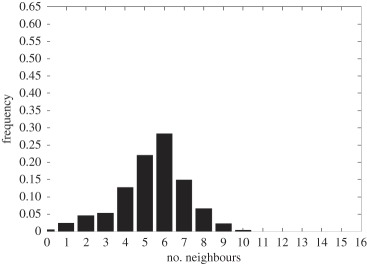	4808.33
5	—	—	—	—
0.5	—	—	—	—
0.05	—	—	—	—
0.005	—	—	—	—

The results show that simulations with parameter values of 0.05 and 0.005 failed. For a value of 0.5, it can already be seen that the cells had unnaturally high numbers of neighbours. Thus, we can assume these parameter values to be unrealistic. By contrast, the image of the cell colony for an endocytosis rate constant of 5 is already very irregular and for values of 50 and 500 the colonies are very loose and extended. After 7 days, only simulations with these high endocytosis rates of 500 and 50 had successfully completed. The colonies in both simulations had grown considerably between day 3 and day 7. Both generally look to be near circular, but they do have areas of extended rows of individual cells. This is mirrored in the distribution of neighbours with about 5% of cells having no, or only one, neighbour.

These simulations show the importance of the endocytosis rate. Without up- or downregulation of the adhesion molecules, the speed of internalization after bond breakage can lead to very compact colonies in which the cells get so close that in a two-dimensional setting, they cannot be individually identified any more. High endocytosis rates, on the other hand, lead to very wide spread colonies with extending strands of cells.

The fact that only such endocytosis rates that are one to three orders of magnitude larger than any of the other intracellular process rates can lead to sustained colony growth seems surprising. This raises the question whether or not the cell interaction processes have been captured in the right way. There are two components, the impact of which we found important to study. Namely, (i) pressure-related entry into G0, which delays the cell cycle, and (ii) the aforementioned constraint of adhesive forces that is not explicitly captured by the intracellular model but results in the cadherin adhesion forces acting as forces preventing cell separation rather than constantly enhancing attraction (referred to as Model 3).

### 4.3. Dynamic adhesion versus dynamic separation forces and the importance of contact inhibition

Following the above argument, we ran simulations with Model 2 with contact inhibition, Model 3 and Model 3 with contact inhibition. Thus, in total three additional sets of simulations were run, in each of which the value of *ρ*_*d*_ was varied between 500 and 0.005 by one order of magnitude at a time. The simulation results for each set after 3 and after 7 days are given in the electronic supplementary material, table S1.

The results in the electronic supplementary material, table S1, show that a very large variation in the behaviour of the cell populations can be observed in the simulations. Some colonies are very compact and cannot grow beyond their original size, whereas others are very loose and show invasive strands of cells. The most obvious overall result is that the combination of both new mechanisms leads to the possibility of observing the behaviour of the cell colonies for the whole range of parameter values, whereas in the other simulations, the cells get too close and cause the code to fail when *ρ*_*d*_ takes on small values.

Based not only on the colony shape and the number of cells, but also excluding simulations that have an extreme distribution of the number of neighbours, the most favourable combination of parameter values and constraints after 3 days is the one used in the last set of simulations, where Model 3 is used with growth inhibition, for a parameter value of 0.5. The results of that simulation are shown in table 5.

**Table 5.** Results of the simulation of a cell population with endocytosis rate 0.5 min^−1^, cell cycle arrest occurs if the pressure in the cell is greater than 13 000 pN and a force equal to the repulsive force in cells closer than their ‘natural state’ (Model 3) after 3 days of real time. (Online version in colour.)

image	no. cells	no. neighbours	average force (pN)
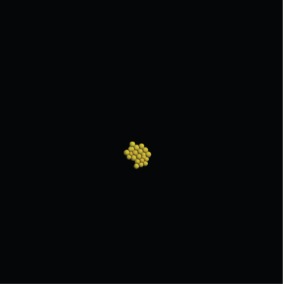	22	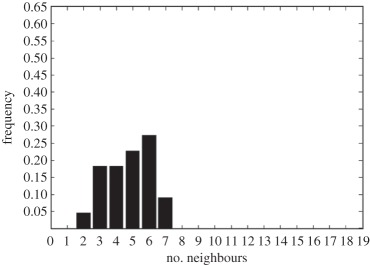	4525.54

After 7 days, most simulations of the three new sets had successfully completed unlike the previous set of simulations of the model with the dynamic adhesion hypothesis (Model 2) alone. In the new set of simulations with Model 2 which included the constraints on cell division, the colonies generated using the parameter values 5, 50 and 500 showed even more irregularity in shape than the results from the set of simulations with Model 2 without contact inhibition. Especially, the colony resulting from using a parameter value of 5 in the simulations shows characteristics of an invasive colony. When comparing it to two-dimensional experimental images of invasive tumour spheroids, a clear similarity can be seen. Such images are for example shown in [23]. Although such experiments are conducted in three dimensions and thus a direct comparison between them and the simulations is not possible, the similarity illustrates that the characteristics of the cells in the model (e.g. a certain endocytosis rate and constantly active adhesive forces) may be related to the phenotypic outcome of genetic changes introduced in the experiment.

In the other simulations of that set, there was no growth between day 3 and day 7 in the simulations that used *ρ*_*d*_ values of 0.5 and 0.05.

In the second set of simulations, where the separation force hypothesis (Model 3) is used, all the colonies look near circular. For the higher parameter values of 500 and 50, a few irregularities can be seen and there are cells that are not in contact with any other cell. However, the simulations resulting from using parameter values of 5 or 0.5, produce suitable configurations and the distribution of neighbours is also similar to those shown in figure 2. The final set of simulations, which combined Model 3 with the mechanism of growth inhibition, shows a very clear decrease in free E-cadherin for decreasing parameter values. Similar to the previous set of simulations without contact inhibition, the overall geometry of the colonies is almost circular for parameter values 0.005, 0.5, 5, 50 and 500. The only real irregular shape can be seen for a value of 0.05 (table 6).

**Table 6.** Results of the simulation of a cell population with endocytosis rate 0.05 min^−1^, cell cycle arrest if the pressure in the cell is greater than 13 000 pN and a force equal to the repulsive force between cells closer than their ‘natural state’ (Model 3) after 7 days of real time. (Online version in colour.)

image	no. cells	no. neighbours	average force (pN)
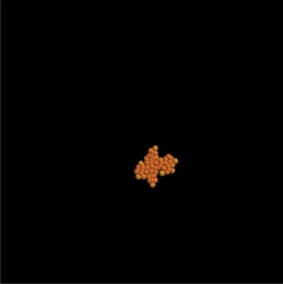	58	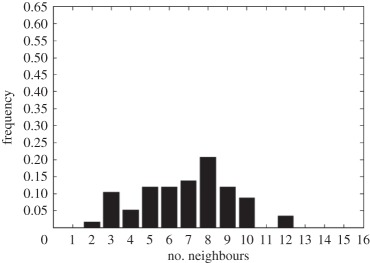	18931.96

However, in both the simulations with a very low E-cadherin endocytosis rate (*ρ*_*d*_ value of 0.005 and 0.05), the amount of free E-cadherin is very low and the colonies show little growth between day 3 and day 7. In addition, the distribution of the number of neighbours the cells have is very different to the ones shown in figure 2. For high endocytosis rates (parameter values of 50 and 500), the circumferences of the cell colonies have slight irregularities and again some cells exist that are not in contact with any other cell. The results of the simulations using the medium rates (parameter values 5 and 0.5) both show relatively circular cell colonies as well as a distribution of the number of neighbours that is comparable to the ones shown in figure 2. The distribution resulting from simulations with *ρ*_*d*_ equal to 0.5, however, gives a slightly better fit to the distributions found from experiments, as the difference between the frequency with which five and seven neighbours are observed is less drastic. There is also less free E-cadherin present in the cells

Given that this combination of parameter value and changes to the original model was also the most favourable in the results of the simulations after 3 days, we examined parameter values around 0.5 more closely. The results of varying *ρ*_*d*_ between 0.1 and 0.9 using increments of 0.1 are given in the electronic supplementary material, table S2. In contrast to the results presented in the electronic supplementary material, table S1, only little variation can be seen between the results of the simulations with the different parameter values.

However, a careful comparison of the distributions of the number of neighbours, the cells have after 7 days shows that the distribution resulting from the simulation with a *ρ*_*d*_ value of 0.6 is closest to the distribution found through experiments in figure 2. This is shown in table 7.

**Table 7.** Results of the simulation of a cell population with endocytosis rate 0.6 min^−1^, cell cycle arrest occurs if the pressure in the cell is greater than 13 000 pN and a force equal to the repulsive force between cells closer than their ‘natural state’ (Model 3) after 3 and after 7 days of real time. (Online version in colour.)

image	no. cells	no. neighbours	average force (pN)
behaviour after 3 days			
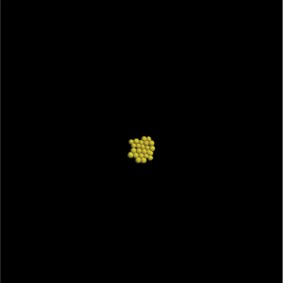	22	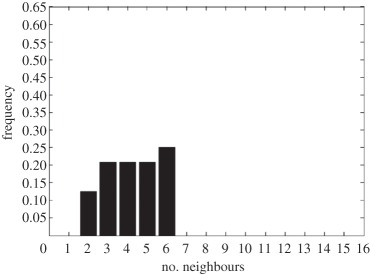	4575.13
behaviour after 7 days			
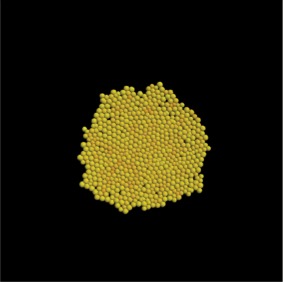	661	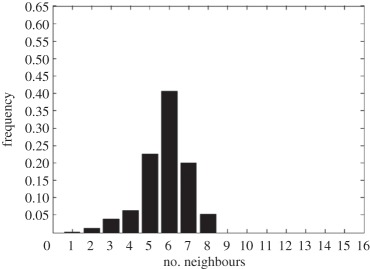	5714.5

The only difference between the distributions is that a small number of cells in the simulation have between one and three neighbours which is lower than what can be seen in the experimental findings. This is most likely due to the fact that in the simulation all the cells are counted, whereas in the experiments only a segment of the inner part of the proliferating epithelial layer is considered. Thus, the low number of neighbours arises most likely from cells at the colony's circumference. This aside, the results of the simulation fit the experimental finding both qualitatively and quantitatively.

**Figure 5.**
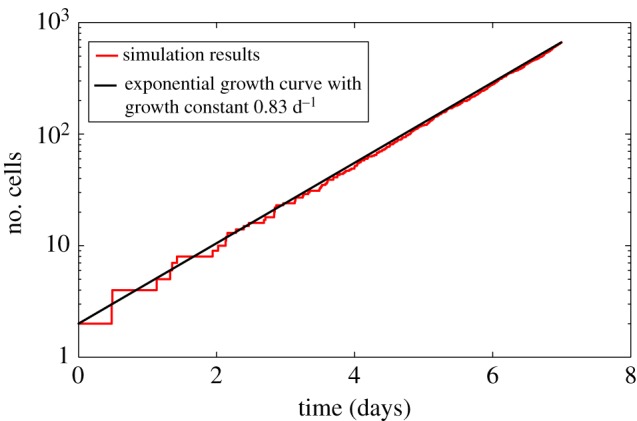
Graph showing the growth of the cell colony arising from the simulation where *ρ*_*d*_ is set to 0.6, cell division is constrained and the force is set to equal the repulsive force between two cells closer than their ‘natural state’ (Model 3). The growth curve of the simulated cell colony is plotted in red (grey in print version). An exponential growth curve with growth rate 0.83 d^−1^ is shown in black. (Online version in colour.)

As mentioned above, we assumed that the cell colony should grow considerably between day 3 and day 7. In this particular simulation, this was clearly the case. In order to get a more precise idea of the emerging growth law, we noted the number of cells against time. The results can be seen in figure 5. The results of the simulation are shown in red (grey in print version). It can be seen that the curve they form resembles an exponential growth curve. When trying to fit an exponential curve to the results by varying the growth constant, we found that the curve of the function4.1
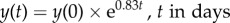
fits the results well giving a doubling time of ln2/0.83 ≈ 20 h. This can be seen in figure 5 where the curve of equation (2.18) is shown in black. This agrees with the well-established assumption that cell monolayers grow exponentially on short timescales [15,24,25]. Given the description in [24] and [15], 7 days are seen to be a short timescale and within the exponential growth regime.

Thus, concerning multiple different measures, this parameter choice and the choice of model changes, show results that fit the data well. This implies that the mechanism of pressure-related entry into G0 as well as the fact that forces between cells are separation and not adhesion forces, are both important cell characteristics, which, together with low endocytosis rates, are necessary to ensure the development of healthy, contiguous cell colonies. It is also important to note that in order to obtain good agreement with experimental findings, the parameters of a simulation have to be chosen where the amount of free E-cadherin is relatively high and the average force at cell–cell contact sites is only just under 6000 pN.

## 5. Discussion

In order to understand how tissues in organisms develop, function and become diseased, it is important to understand how cells interact within these tissues. To complement experimental work which studies cell behaviour, interactions and cell colony development in two-dimensional tissue cultures and bridge the gap between these experiments and individual force measurements, we have developed a multi-scale computational model which captures all of these processes. The model is based on previous work by Ramis-Conde *et al.* [5] and takes into account new mechanisms of cell–cell interaction, such as cell-cycle arrest due to pressure and a transition from an adhesion to a separation force framework. It also includes the spatial aspect of intracellular organization.

Some very interesting points were highlighted by trying to parametrize the intracellular E-cadherin–*β*-catenin pathway through fitting the simulation results to literature data. Experimentally, the adhesion between cells is generally measured by dual-pipette assays [4,26,27]. Owing to their set-up, it is only possible to measure the force generated between two cells during separation processes. Thus, it is unknown whether the force is the same between two cells in isolation and two cells in a cell colony. Here, we investigated both possibilities: (i) the force between two cells is always the same due to a limitation of the number of E-cadherin bonds that can form between two cells and (ii) the force between two cells decreases during the establishment of adhesion bonds with further neighbouring cells due to a redistribution of E-cadherin–*β*-catenin complexes. We found that only a model using the latter assumption could be parametrized such that it would fit the separation force data by Chu *et al.* [4] well. This implies that the adhesion between cells in a monolayer is much less than that between two individual cells with the force decreasing with the number of cells that are attached to one another. This is a very interesting hypothesis and although to our understanding currently difficult, it would be very interesting to devise experimental techniques to test this. Dynamic E-cadherin production and degradation do of course give further possibilities for the development of bonds between cells in colonies. Whether or not this would have an impact on the hypothesis developed here is beyond the scope of this study but it will be interesting to include it in future work. One possible way to extend this aspect of the current model would be to develop the ‘point attachment model’ of Hammer & Lauffenburger [28]. This model considered receptor-mediated adhesion in cells which were attached to a substrate and experiencing shear flow. The model focused on the receptor–ligand interactions taking place in the contact area between an individual cell and the substrate the cell was attached to. This idea could be extended to model the interactions between two cells in our model as a quasi-chemical-activated decoupling process.

We further investigated the influence of the rate of E-cadherin–*β*-catenin endocytosis and complex disruption after bond breakage on cell colony development. However, only those simulations could successfully run over 7 days of real time, which had endocytosis rates of 50 and 500 min^−1^ which is one to three orders of magnitude higher than any of the other intracellular rate parameters. Thus, we decided on some modifications of the model. These modification were, firstly, a pressure threshold for the progression through the cell cycle of 13 000 pN and, secondly, a shift from interpreting the experimental measurements as adhesion data to seeing them as separation force data. Both of these modifications are biologically sensible and, as they allowed colony growth for lower E-cadherin endocytosis rates, most likely necessary to model the development of colonies in two-dimensional cultures realistically. This implies that both, the pressure-related cell cycle arrest and the fact that the forces between cells are separation and not adhesion forces, may be important cell characteristics in order for healthy, contiguous cell colonies and tissues to develop.

The simulations we conducted with and without these modifications showed that these characteristics as well as the speed at which E-cadherin–*β*-catenin complexes are internalized after bond-breakage could have a large influence on the integrity of cell colonies independent of E-cadherin expression levels. High rates led to quickly growing colonies with low adhesion levels, rough surfaces and extending strands of cells as is seen in invasive tumours *in vivo* and *in vitro* [23]. Very low rates led to tight clumps of cells that did not grow significantly over any length of time. This highlights that independent of expression levels of E-cadherin or other adhesive molecules, a wide variety of cell colony patterns can develop based on cell-specific kinetic rates and phenotypic characteristics. It also highlights how the model may be used to understand the cell phenotypic changes brought about by genetic mutations, the effects of which can only be seen at the tissue level.

Finally, we found an endocytosis rate constant which led to cell colonies that followed an exponential growth regime as has been shown for healthy epithelial colonies experimentally [15,24,25]. These colonies also showed a distribution of the number of neighbours as it has been measured experimentally for growing epithelial layers. Surprisingly, the average adhesion/separation force between a pair of cells in these colonies was much lower than has been measured between two cells [4]. Even extrapolating this force to a cell that has more than one neighbour and taking the redistribution of E-cadherin–*β*-catenin complexes between adhesion sites into account, one would expect a much higher cell–cell adhesion/separation force and very little free E-cadherin. To our knowledge, there are unfortunately no data in the available literature that we can compare this with as, although experiments that use fluorescence staining of E-cadherin exist, they are difficult to quantify. Thus, this comparatively low separation force in cells in the epithelial monolayer compared with the separation force of two individual epithelial cells can only be hypothesized to exist and it emphasizes again that it would be interesting to devise experiments that can test this. This also emphasizes the point that the results of adhesion measurements that have been conducted with dual-pipette assays on a pair of cells might not transfer easily to cells which are in contact with a greater number of neighbouring cells in monolayers and tissues

The model developed here is a very general model of cell colonies in two-dimensional cultures. Thus, it was possible also to find general results which showed that it may not be possible to infer events at the tissue level from individual cell experiments due to additional mechanisms and different biophysical properties in multi-cell systems compared with individual cells. This will hopefully inspire experimentalists to study these differences in order to deepen the understanding of the interactions of cells in tissues and of how events at the single-cell-level transfer to the tissue level. The model developed here may help with these investigations by making it possible to infer cell phenotype from observed tissue level behaviour of genetically modified cells. Furthermore, a lot of specific experiments could be mimicked by including chemical gradients or other environmental changes as well as special characteristics of the cells under consideration. With that kind of specific input data much more specific results than the hypotheses developed here could be found. Such a model could then help to back up experimental results as well as explore possible experimental outcomes on a faster timescale than is possible in the laboratory. For further investigations into the development of invasive cell colonies as is seen in tumour cell lines, this model can also be coupled to a model of cell migration on extracellular matrix fibres as we have developed previously [20]. This may further help to understand the transition to invasiveness in tumour cell lines and primary cells and the role the extracellular matrix plays.

## Supplementary Material

Electronic Supplementary Material (ESM) for “Multi-scale modelling of the dynamics of cell colonies: insights into cell adhesion forces and cancer invasion from in silico simulations”
