# Supplementary material for: Multi-scale modelling of the dynamics of cell colonies: insights into cell-adhesion forces and cancer invasion from in silico simulations
Source: J R Soc Interface. 2015 Feb 6;12(103):20141080. doi: 10.1098/rsif.2014.1080 (PMC4305411; doi:10.1098/rsif.2014.1080)
Supplement: Electronic Supplementary Material (ESM) for “Multi-scale modelling of the dynamics of cell colonies: insights into cell adhesion forces and cancer invasion from in silico simulations” [file rsif20141080supp1.pdf]

Electronic Supplementary Material (ESM) for "Multi-scale modelling of the dynamics of cell colonies: insights into cell adhesion forces and cancer invasion from in silico simulations"

Daniela K. Schlüter[1]*, Ignacio Ramis-Conde[2] and Mark A. J. Chaplain [1]

[1] Division of Mathematics, The University of Dundee, Dundee, Scotland;

[2] Universidad de Castilla la Mancha, Department of Mathematics, Faculty of Education, Cuenca, Spain

1 Model dynamics

To investigate the dynamics of the intracellular model we arrived at after the parameters estimations and its capabilities as part of a multi-scale model further, we ran simulations in which we tracked the intracellular dynamics as well as the cells during the process of initial contact and bond formation. The results are shown in Figure S1. The top row of plots shows the development over 40 minutes at the cellular level. In the figures it can be seen how the positions of the cells relative to each other changes until a steady-state configuration is reached where the repulsive and adhesive forces are in balance. At the same time these plots show the amount of E-cadherin that is taken up in bonds at the cell-cell contact site - the intensity of the yellow colour in the otherwise red cells is proportional to the amount of E-cadherin in the cytosol. Thus the redder the image of the cell is, the less E-cadherin is in the cytosol and the more E-cadherin is at the cell-cell contact site. The time course of the E-cadherin dynamics is also shown in the plots in the bottom row of the figure. Here the dynamics of the intracellular components in both cells are shown over 120 minutes. It can be seen that the dynamics are the same in both cells. After initial complex formation and a fast increase of E-cadherin–β-catenin complexes at the contact site, the dynamics slow down to reach a steady-state. These simulations show that the intracellular dynamics are well-modelled in the cell level model and provide a multiscale model of cell-cell interaction dynamics.

To investigate the adhesion behaviour at an intracellular level in highly populated regions of cellular layers we performed simulations for six cells consecutively attaching to each other in a hexagonal packing configurations. We also considered scenarios where different numbers of

*Current address: CHICAS, Medical School, Lancaster University, Lancaster, United Kingdom

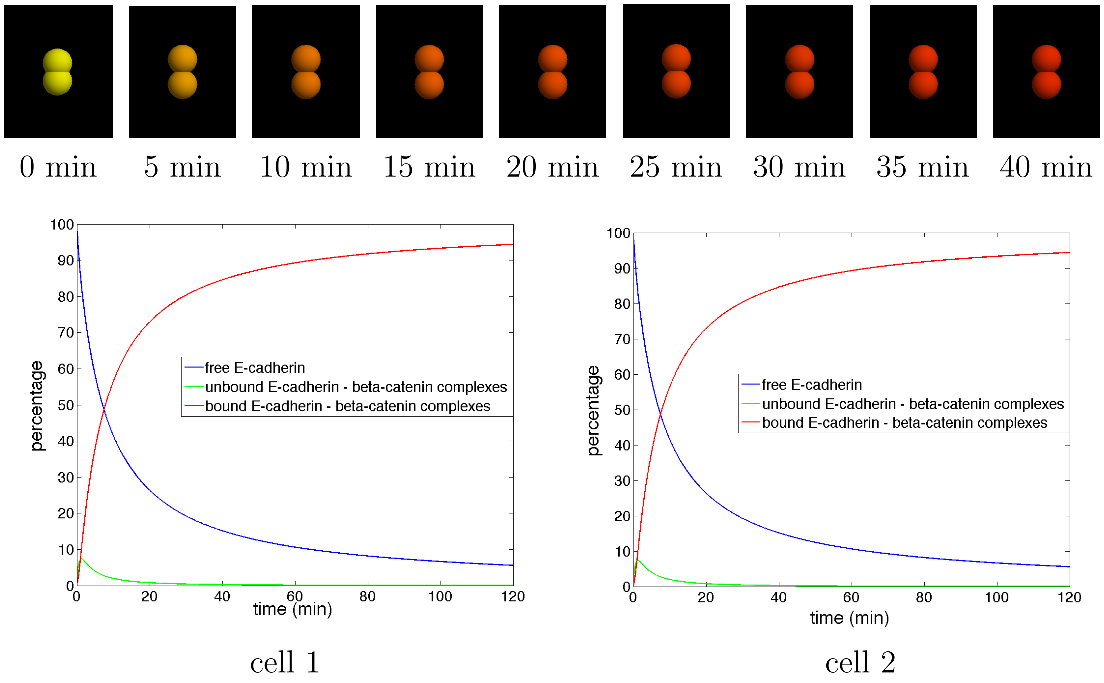

Figure S1: Figures showing the time course development of the multiscale cell model at the cell as well as the intracellular level in a simulation with two cells. In the top row of plots, E-cadherin in the cytosol is shown by the intensity of the yellow colour. Thus the uptake of E-cadherin in cell-cell bonds can be followed over time through the colour change of the cells in the figure from yellow to red. At the same time it can be seen that the distance between the mid-points of the two cells changes until the two cells are at a steady-state configuration. The bottom row of plots shows the intracellular dynamics in both cells during these simulations.

cells attached themselves and detached themselves from one cell. The simulation results are shown in Figure S2 in terms of the force between the cell of interest and its neighbours. Figure S2(a) shows the force at different contact sites during and after the consecutive attachment of six cells to the cell of interest. It can be seen that independent of the length of time between attachments, the E-cadherin–β-catenin complexes were redistributed successfully such that the forces were equal at all cell-cell contact sites at the end of the simulation. Figure S2(b) shows the forces at different contact sites during and after attachment and detachment processes as well as the detachment of two cells simultaneously. Initially all six neighbours were attached to the cell of interest. After 30 minutes we forced one of the cells to detach itself and it can be seen in the plots that the E-cadherin–β-catenin complexes that had been forming the bonds at this cell-cell contact site, were internalised and then recycled to enforce the other cell-cell attachments. This becomes clear as the graphs depicting the force at the different cell-cell contact sites show a step like increase in their values. The same can be seen for further cell detachments after different time intervals. After the detachment of 4 cells (cells 3-6), 80 minutes into the simulation, we forced cell number 3 to reattach itself to the cell of interest and the graphs show the expected behaviour of the redistribution of force. At 110 minutes, we forced two cells to detach themselves and again, the graph of the final neighbour left, shows a smooth increase in force at this cell-cell contact site.
These simulations show that the model exhibits the behaviour one would expect to see at

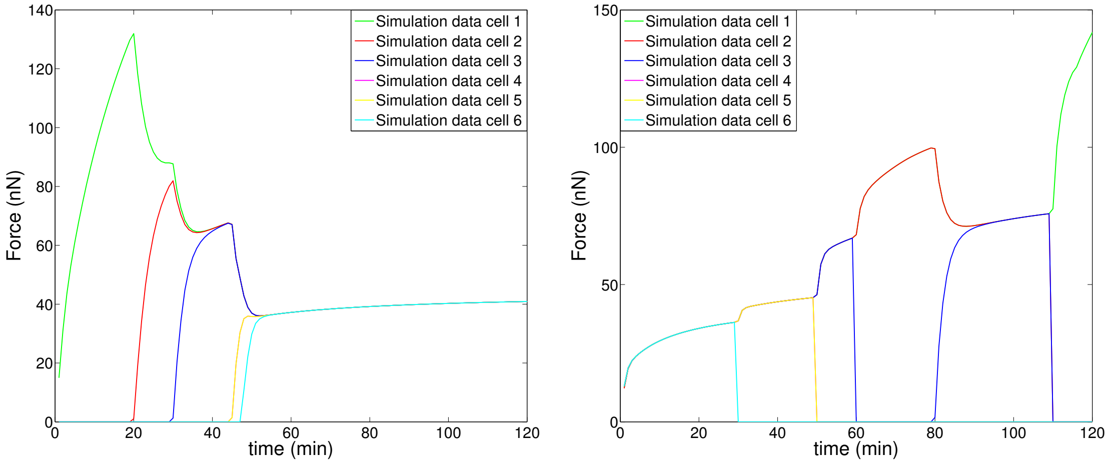

(a) (b)

Figure S2: Graphs showing the time course of the forces at different cell–cell contact sites after multiple cell–cell attachment and detachment processes. Plots in figure (a) show the forces at different contact sites during and after the consecutive attachment of six cells to the cell of interest. Plots in figure (b) show the forces at different contact sites during and after attachment and detachment processes as well as the detachment of two cells simultaneously.

cell-cell contact sites during and after attachment and detachment processes. It also fits the cell-cell adhesion data by [1] well and is stable in a variety of scenarios.

2 Cell Colony simulation results

For the investigation of the effect of separation forces rather than adhesion forces acting between cells (Model 3 rather than Model 2) and pressure-related regulation of proliferation (contact inhibition), multiple sets of simulations were run. One set of simulations was run with Model 2 (the dynamic adhesion hypothesis) and contact inhibition of proliferation, one set was run with Model 3 (the dynamic separation force framework) and one final set with both Model 3 and contact inhibition of proliferation. In each one of these we varied the value of ρ_d between 500 and 0.005 by one order of magnitude at a time. The first set we called *'contact inhibition'*, as here the division of a cell was constrained by the pressure it was under. The second set has the name *'separation force framework'* to note that in these simulations Model 3 was used such that the force was set to be equal to the repulsion in cells closer than their natural state. Finally the third set is called *'separation force framework and contact inhibition'* to note that here both changes were made. The results, first after three days and then after seven days, are shown in Table S1. Whenever a row does not have any entries, that means that those simulations failed due to the cells getting too close. The colours of the cells in the plots in the second column of the table, are related to the amount of free E-cadherin which is shown in yellow in the otherwise red cells.

Table S1: Table showing the results of varying ρ_d, the constraints under which cells can enter M-phase and the force that cells generate between them if they are closer than their natural state.

ρ_d	Image	#cells	#neighbours	average force
**Behaviour after 3 days**				
	contact inhibition			
500	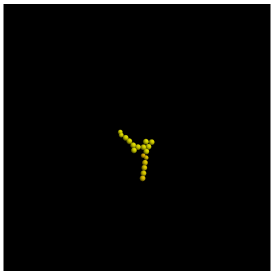	18	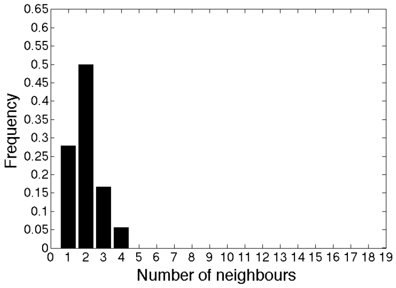	0pN
50	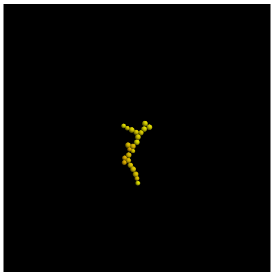	23	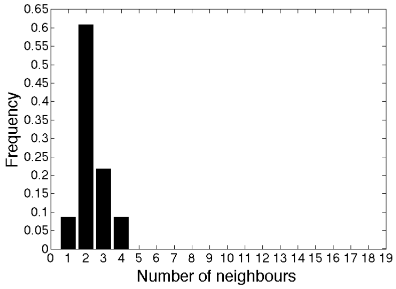	13502.69pN
5	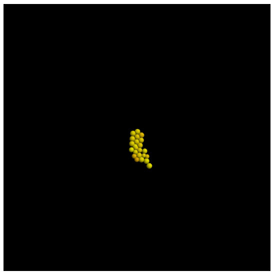	23	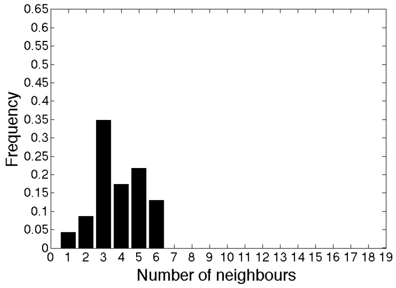	9857.29pN
0.5	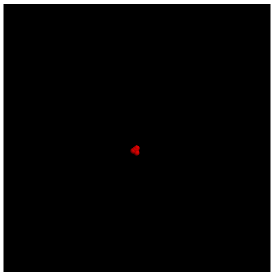	6	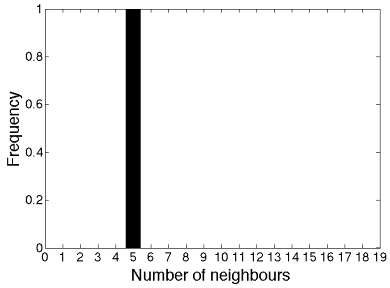	52154.77pN

Table continues on next page...

...Table continued from previous page.

ρ_d	Image	#cells	#neighbours	average force
0.05	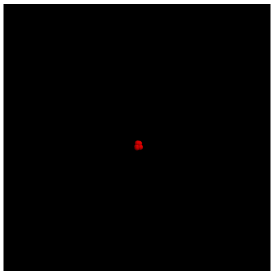	5	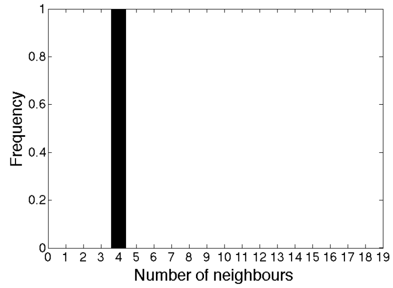	65414.28pN
0.005	–	–	–	–
	separation force framework			
500	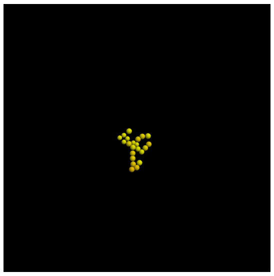	22	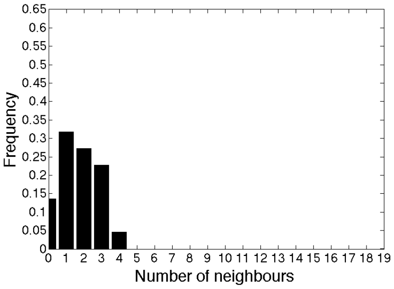	3079.32pN
50	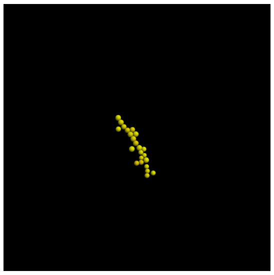	24	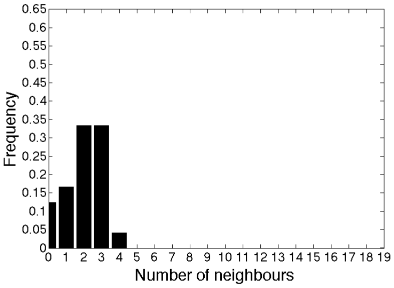	0pN
5	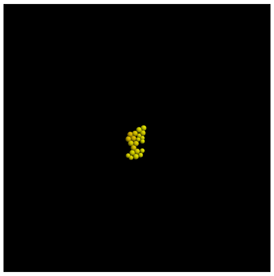	21	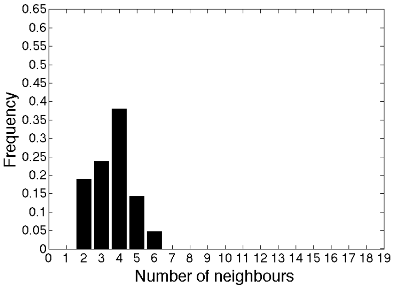	323.56pN

Table continues on next page...

...Table continued from previous page.

ρ_d	Image	#cells	#neighbours	average force
0.5	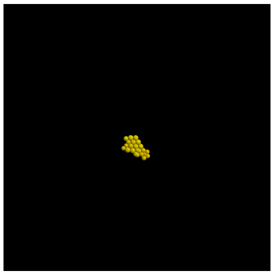	24	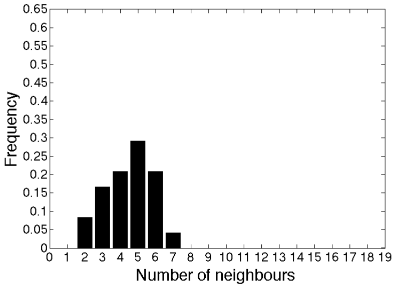	5071.39pN
0.05	–	–	–	
0.005	–	–	–	–
	separation force framework and contact inhibition			
500	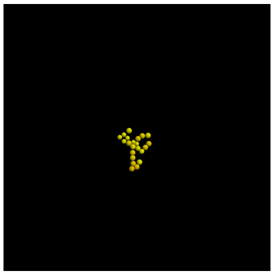	22	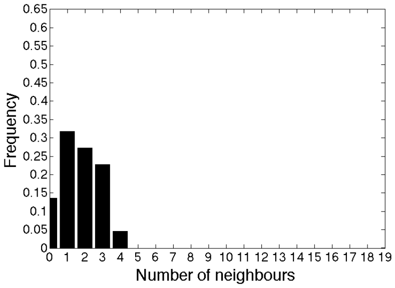	3079.32pN
50	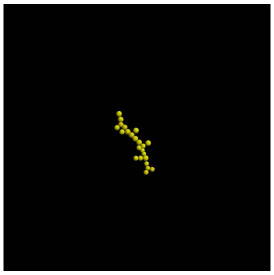	24	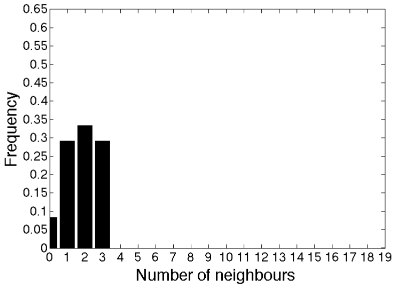	6296.73pN
5	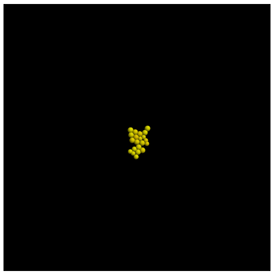	21	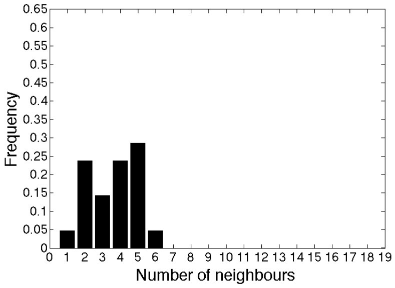	3782.98pN

Table continues on next page...

...Table continued from previous page.

ρ_d	Image	#cells	#neighbours	average force
0.5	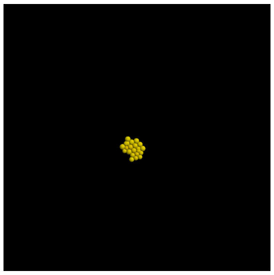	22	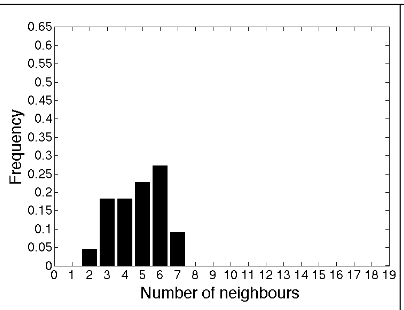	4525.54pN
0.05	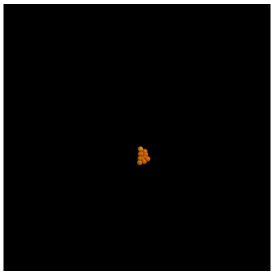	11	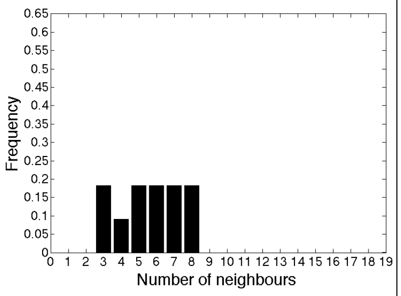	20528.58pN
0.005	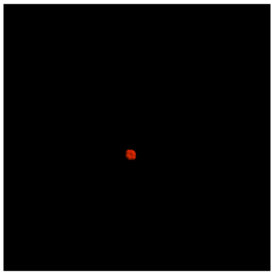	7	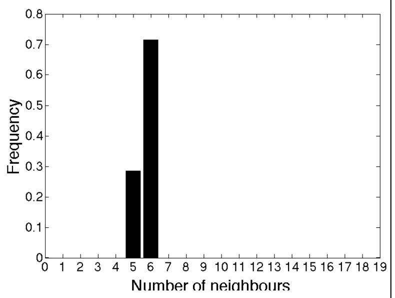	37182.90pN
**Behaviour after 7 days**				
	contact inhibition			
500	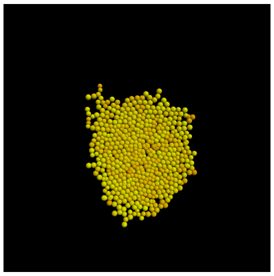	560	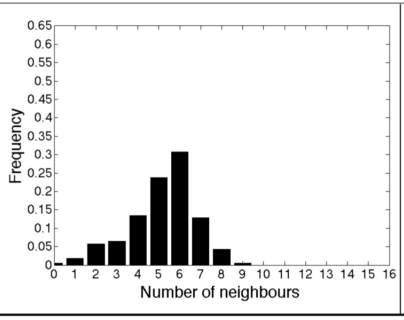	5080.04pN

Table continues on next page...

...Table continued from previous page.

ρ_d	Image	#cells	#neighbours	average force
50	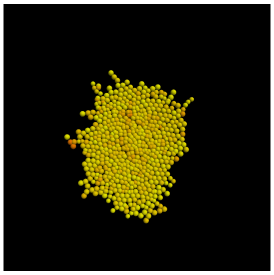	648	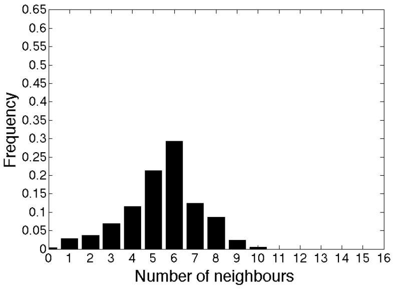	5167.65pN
5	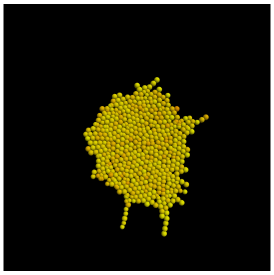	613	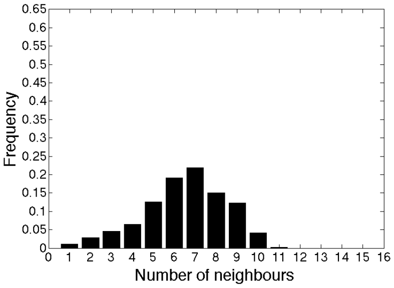	4611.23pN
0.5	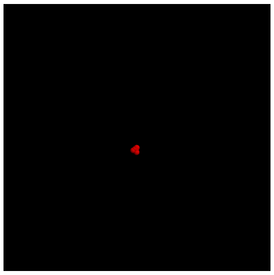	6	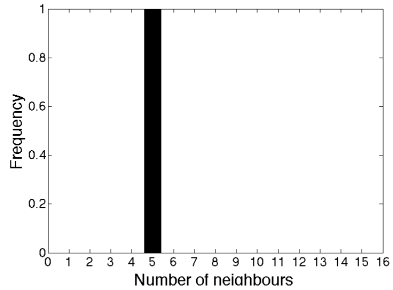	52109.05pN
0.05	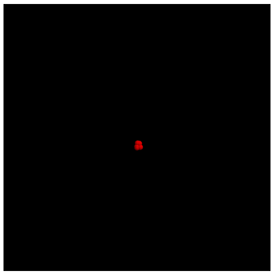	5	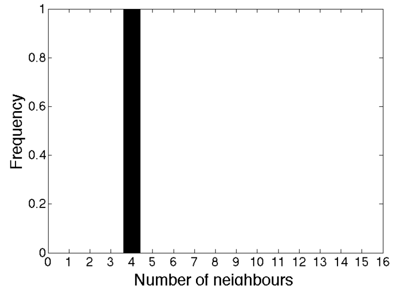	65454.69pN
0.005	–	–	–	–

Table continues on next page...

...Table continued from previous page.

ρ_d	Image	#cells	#neighbours	average force
	separation force framework			
500	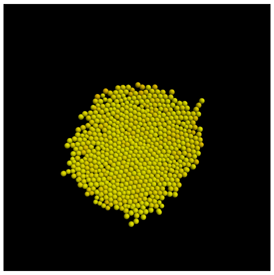	659	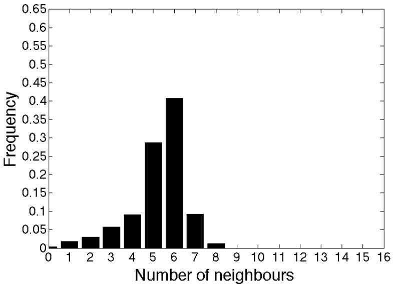	1770.12pN
50	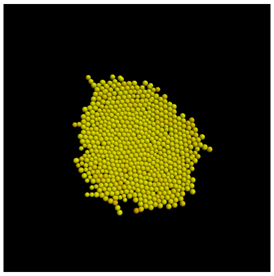	650	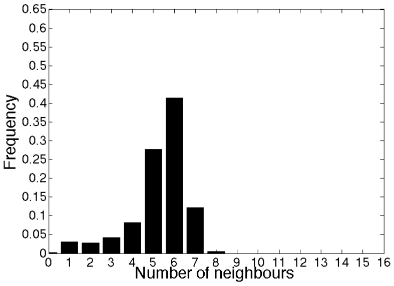	1723.86pN
5	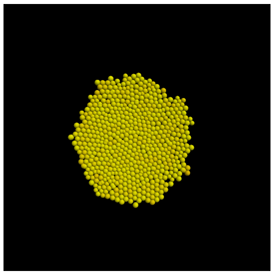	631	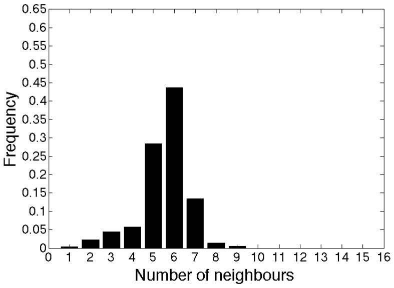	1956.04pN
0.5	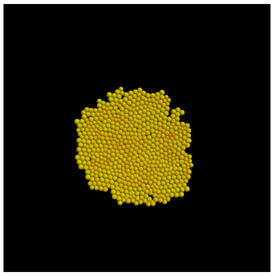	640	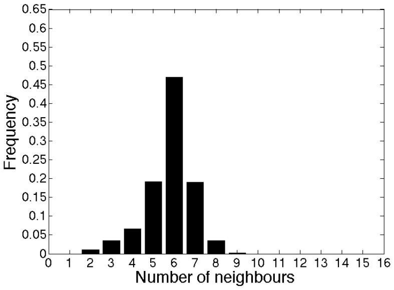	6680.74pN
0.05	–	–	–	
0.005	–	–	–	–

Table continues on next page...

...Table continued from previous page.

ρ_d	Image	#cells	#neighbours	average force
	separation force framework and contact inhibition			
500	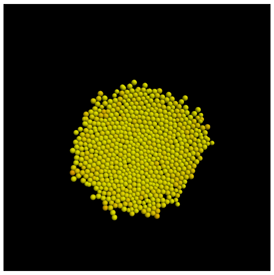	659	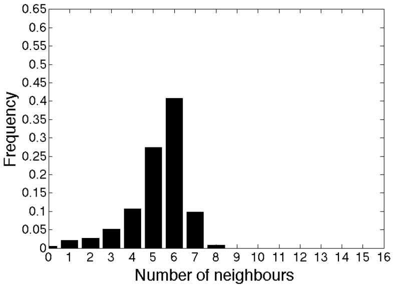	1783.20pN
50	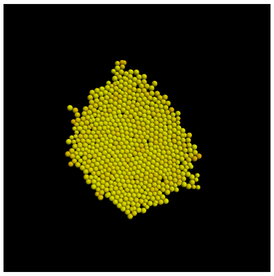	639	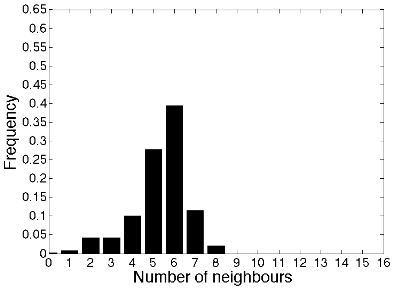	1937.0pN
5	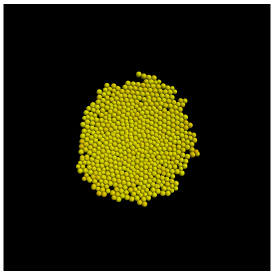	597	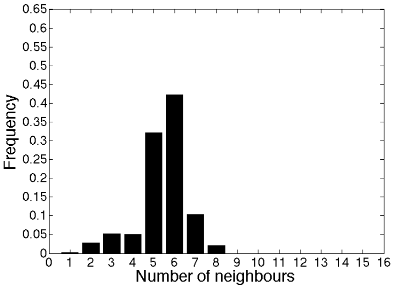	1972.87pN
0.5	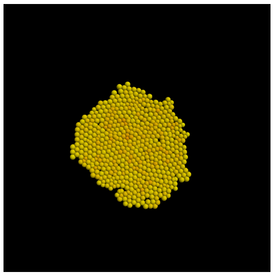	581	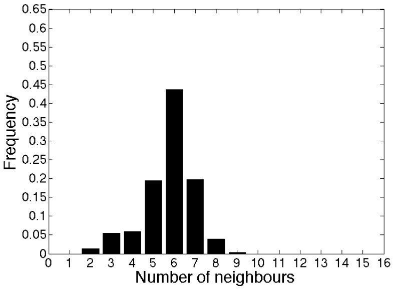	6392.6pN

Table continues on next page...

...Table continued from previous page.

ρ_d	Image	#cells	#neighbours	average force
0.05	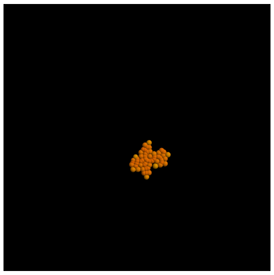	58	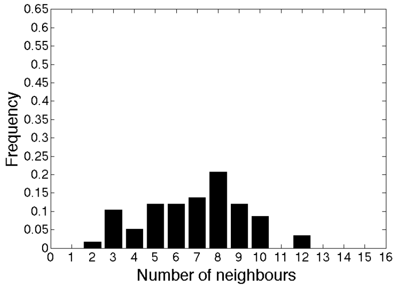	18931.96pN
0.005	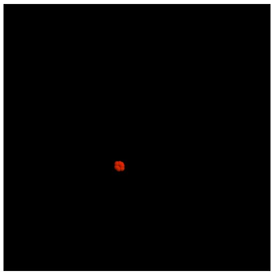	7	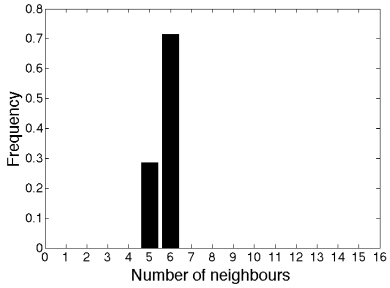	37380.47pN

Given that the combination of an endocytosis rate of 0.5 with the model that included the separation force framework and contact inhibition of proliferation was the most favourable in the results after three and after seven days, we examined parameter values around 0.5 more closely again, using the pressure regulated entry into G0 as well as assuming a force equal to the repulsive force if cells get closer than their assumed natural state. The results of varying ρ_d between 0.1 and 0.9 using increments of 0.1 are given in Table S2.

Table S2: Table showing the results of varying ρ_d between 0.1 and 0.9 with increments of 0.1 and using both, constraints on cell division and a force equal to the repulsive force if cells get closer than their assumed natural state

ρ_d	Image	#cells	#neighbours	average force
**Behaviour after 3 days**				
0.9	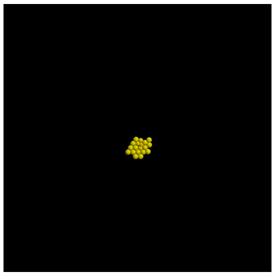	19	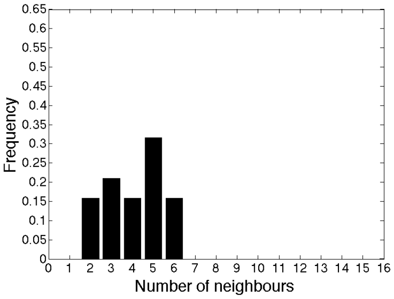	3553.33pN
0.8	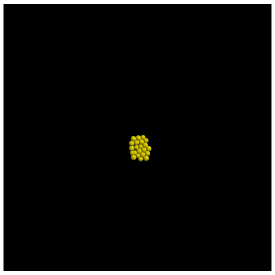	22	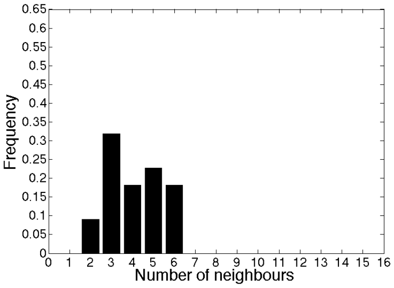	3970.7pN
0.7	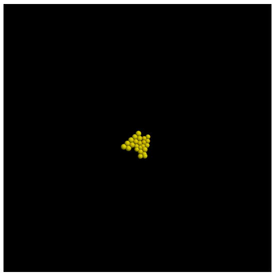	24	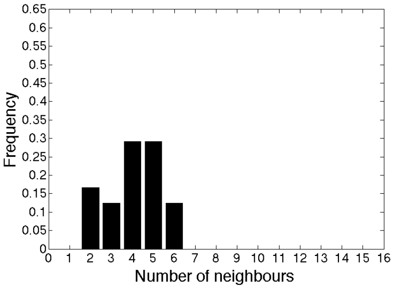	4000.27pN

ρ_d	Image	#cells	#neighbours	average force
0.6	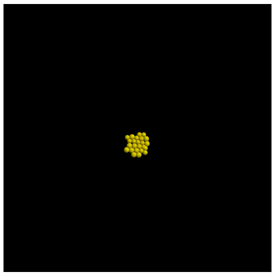	22	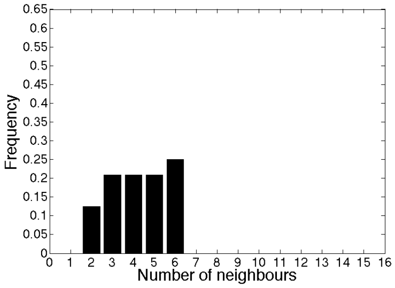	4375.13pN
0.5	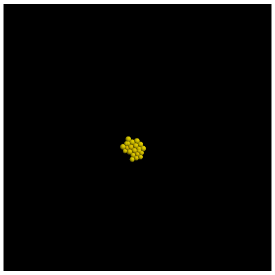	22	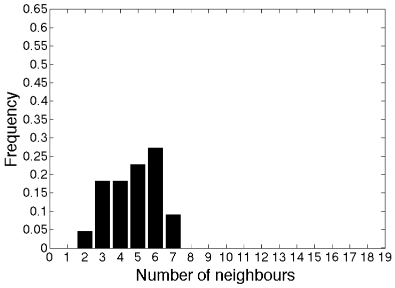	4525.54pN
0.4	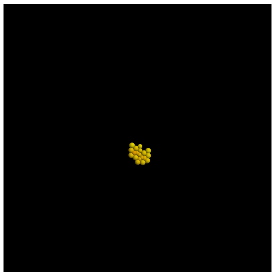	19	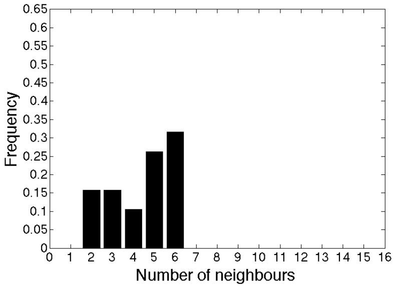	5500.54pN
0.3	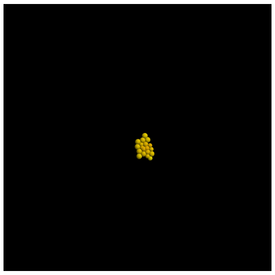	19	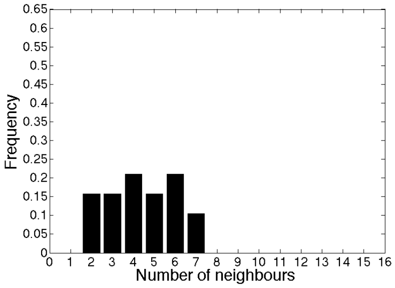	5920.43pN
0.2	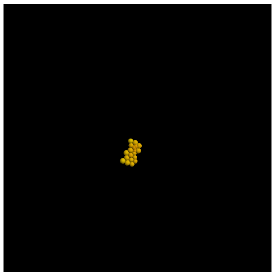	19	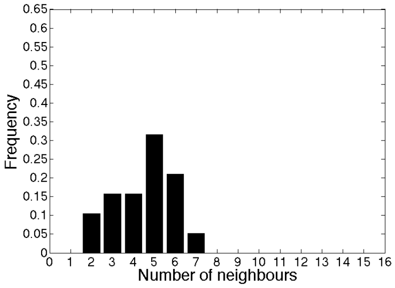	8250.23pN

ρ_d	Image	#cells	#neighbours	average force
0.1	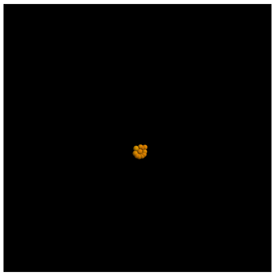	12	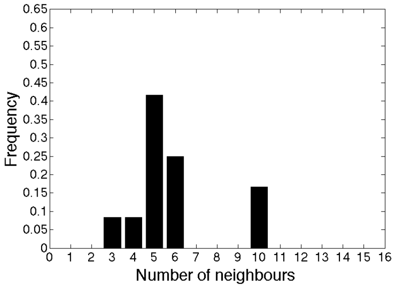	15786.24pN
**Behaviour after 7 days**				
0.9	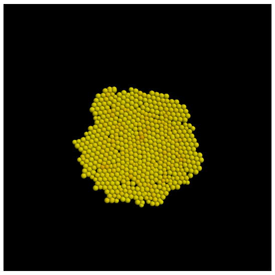	594	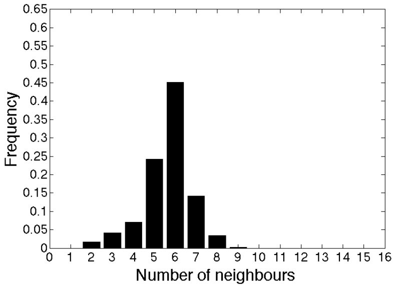	3898.86pN
0.8	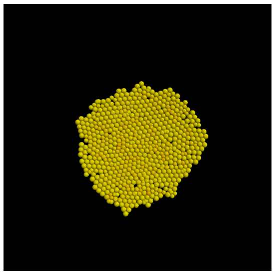	647	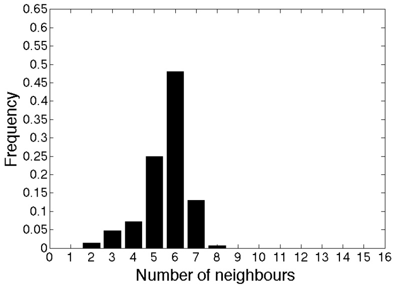	4503.34pN
0.7	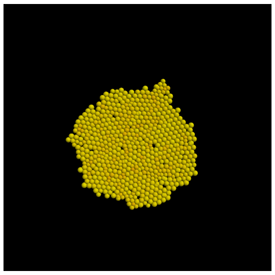	662	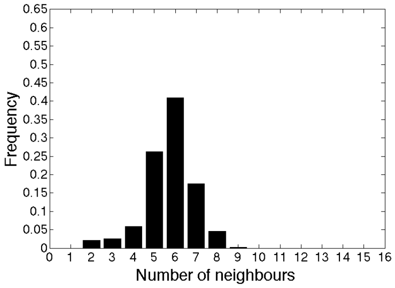	4905.28pN
0.6	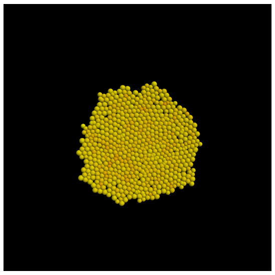	661	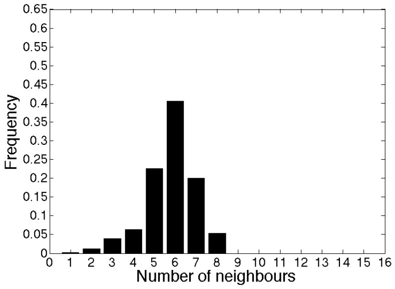	5714.5pN

ρ_d	Image	#cells	#neighbours	average force
0.5	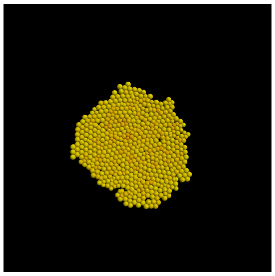	581	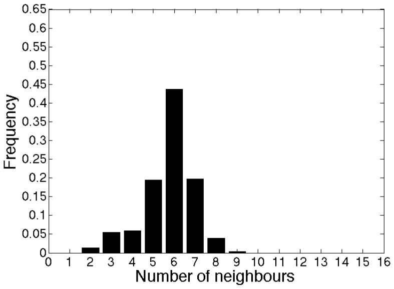	6392.59pN
0.4	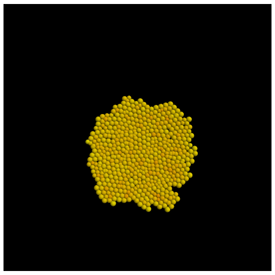	569	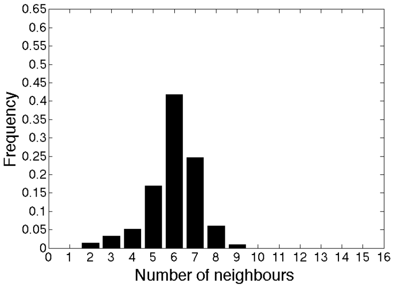	7413.07pN
0.3	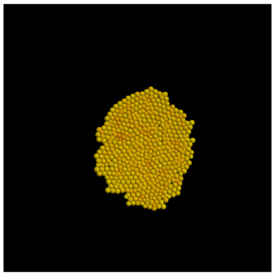	543	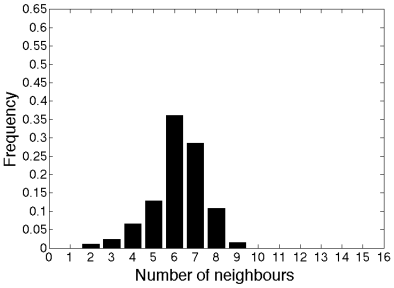	8672.28pN
0.2	–	–	–	–
0.1	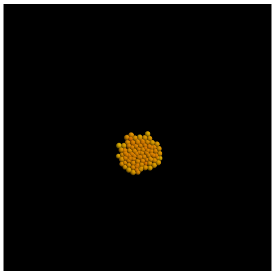	93	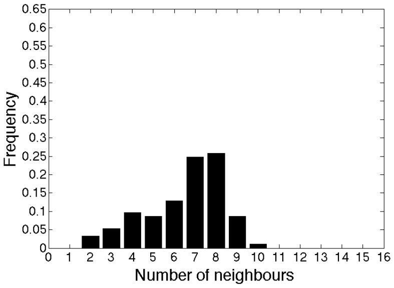	14794.69pN

Supporting References

[1] Chu YS, Thomas WA, Eder O, Pincet E, Thiery JP, Dufour S. Force measurements in E-cadherin–mediated cell doublets reveal rapid adhesion strengthened by actin cytoskeleton remodeling through Rac and CdC42. J Cell Biol. 2004;167:1183–1194.
